# Changes in physical activity during the retirement transition: a theory-based, qualitative interview study

**DOI:** 10.1186/s12966-015-0186-4

**Published:** 2015-02-21

**Authors:** Suzanne McDonald, Nicola O’Brien, Martin White, Falko F Sniehotta

**Affiliations:** Institute of Health & Society, Newcastle University, Baddiley-Clark Building, Richardson Road, NE2 4AX Newcastle uponTyne, UK; Fuse, UKCRC Centre for Translational Research in Public Health, Newcastle upon Tyne, UK

**Keywords:** Physical activity, Retirement, Qualitative research, Behaviour change, Theory Domain Framework

## Abstract

**Background:**

There are considerable inter-individual differences in the direction and degree of change in physical activity (PA) levels during the retirement transition. There is currently a limited theoretical understanding of how these differences can be explained. This study aimed to explore and compare perceptions about how theory-based factors influence PA change during the transition from employment to retirement among individuals approaching retirement and recently retired.

**Methods:**

Theory-based, one-to-one, semi-structured interviews were conducted with a purposive sample of 28 adults (15 retired) within 24 months of retirement. Participants were sampled to reflect a diverse range of socio-economic and occupational backgrounds. The interview was based on the 12 domains within the Theory Domain Framework and designed to elicit anticipated or experienced retirement-related changes in PA behaviour and perceived determinants. Interview transcripts were analysed using Framework analysis to explore intra- and inter-individual perceptions of how PA changes after retirement and the factors which may influence this change.

**Results:**

The majority of participants perceived retirement to be related to an increase in PA levels. Four themes emerged from the data regarding factors perceived to influence changes in PA behaviour after retirement: (1) resources for PA; (2) structure of daily life in retirement; (3) opportunities for PA; and (4) transitional PA phases after retirement. Retirement is associated with a number of inter-related changes and opportunities which can have a positive or negative impact on PA behaviour. The influence of these factors does not appear to be static and may change over time. A number of different transitional phases may be experienced after leaving work and each phase may have a differential impact on PA behaviour.

**Conclusions:**

The findings of this qualitative study contribute to the theoretical understanding of PA change during the retirement transition. Each post-retirement PA trajectory is highly individual and personalised intervention approaches to increase PA during the retirement transition may be most successful. Future research should focus on the maintenance of PA change during the retirement transition and should develop and evaluate interventions to promote and maintain PA during retirement.

**Electronic supplementary material:**

The online version of this article (doi:10.1186/s12966-015-0186-4) contains supplementary material, which is available to authorized users.

## Background

Adults aged 60 years and over represent the fastest growing segment of the population [1]. As a result, the prevalence of age-related chronic disease and disability is increasing, placing considerable pressure on health care services. Physical activity (PA) is among the most important, modifiable factors influencing the ageing trajectory. There is compelling evidence that participation in regular PA can prevent or delay the onset and progression of several chronic diseases and contribute towards a reduction in physical and cognitive functional decline in old age [2-4]. Despite the well-documented benefits of PA for healthy ageing, PA levels have been shown to decline considerably as age increases [5].

Previous cross-sectional and longitudinal studies provide evidence that PA levels change during the transition from employment to retirement [6-11]. However, there is a lack of conclusive evidence regarding the direction and degree of post-retirement PA change. For example, retirement has been associated with an increase in PA levels in some large longitudinal studies but with a net decrease in others [7-9]. A systematic review of studies examining changes in PA across the retirement transition demonstrated that socio-economic status (SES) moderated the effects of retirement on PA levels [12] which may potentially explain these inconsistencies. Individuals who retired from manual occupations showed a decrease in PA levels, whilst individuals who retired from non-manual occupations showed a maintained or increased level of PA after retirement.

In order to understand why retirement is associated with changes in PA levels, a previous systematic review synthesised the findings from qualitative studies exploring the experiences of and views on PA reported by people in retirement [13]. The review concluded that a number of key motives and challenges associated with PA in retirement could be interpreted as potential explanations of why PA levels increase or decrease after retirement, respectively. The main motives for PA in retirement included a heightened awareness of ageing, which underscored the importance of PA for health and well-being; having a lifelong history of engaging in PA behaviour, which continued into retirement; and broader benefits such as PA providing a personal challenge, opportunities to socialise and using PA to create a daily structure in post-retirement life. Lack of time and low value placed on engaging in PA, particularly in people who retired from manual occupations, were key challenges that were attributed to decreased PA levels after retirement.

It is important to understand individual perceptions about the reasons for PA change during the retirement transition from a theoretical perspective. This can help to link qualitative evidence to previous research on the causal determinants of behaviours and to develop a better understanding of viable targets for future interventions [14]. Only two studies identified in the systematic review of qualitative studies [13] referred to specific theoretical explanations for PA behaviour in retirement [15,16]. One study used semi-structured interviews based on self-determination theory [17] and habit theory [18] to explore differences in motivation and decision-making processes between individuals who were either physically active or inactive in retirement [15]. The findings suggested that social factors, lifelong PA habits and seeking a sense of purpose were common motivations for engaging in PA after retirement. A second study [16] referred to the Oxford Model of Sports Participation [19], a theoretical model of sporting behaviour change, which hypothesises that a number of neighbourhood and individual variables determine population engagement in sports and exercise behaviour. In this study, attitudes, opportunities, motivators and barriers to sports and exercise were explored in focus groups comprising individuals who had been retired for at least 6 months and a small number of individuals who were self-classified as ‘semi-retired’. The findings emphasised a number of key motivators and barriers to participant’s current sports and exercise behaviour including the physical, mental and social benefits of PA and physical limitations preventing PA, respectively.

Only a limited number of theory-based factors have been investigated as potential candidates explaining retirement-related PA changes, meaning that the theoretical basis of PA change during the retirement transition remains poorly understood. Furthermore, none of the primary studies included in the systematic review explicitly explored how PA is perceived to change during the transition from employment to retirement and did not explore whether these views differed between individuals at different stages during the transition. Previous studies have focused mainly on retired individuals who have been retired for several years. Such retrospective research may be susceptible to recall bias. It is currently unclear how individuals who are approaching or who have recently experienced retirement perceive PA to change during this transition and the factors which may be responsible for this change. During this transition individuals may be undergoing several significant lifestyle changes which may represent important factors that determine PA change after retirement.

The Theory Domain Framework (TDF) [20] provides a broad and inclusive approach to understanding theory-based predictors of behaviour and behaviour change. The TDF was developed by an expert consensus group, which reviewed 33 behavioural theories and their associated 128 theoretical constructs. The consensus group suggested 12 theoretical domains based on commonalities between constructs. Therefore, the TDF broadly covers all scientific explanations of behaviour and behaviour change based on current theorising. The TDF provides a valuable framework for identifying potentially relevant influences on behaviour which can be mapped to existing theory. The TDF has been used to examine the perceived determinants of a wide range of health-related behaviours including the difficulties implementing smoking cessation advice among midwives, help-seeking behaviour in stroke witnesses, adherence to behavioural changes in a lifestyle intervention for type 2 diabetes, and physical activity in stroke survivors [21-24].

This study aimed to investigate the influence of a broad set of factors on PA change during the retirement transition. Whether these perceptions differed as a function of expectation or experience of retirement was also explored.

## Methods

### Design

One-to-one semi-structured interviews were conducted using open-ended questions and a pre-specified interview schedule based on the TDF [20].

### Participants

A purposive sample of adults within 24 months pre- and post-retirement was recruited between June 2011 and August 2012 from voluntary organisations, community advertisements and by word of mouth. Study inclusion was limited to individuals within 24 months pre- or post-retirement in an attempt to elicit accurate representations of the impact of retirement on PA behaviour. The definition of retirement was based on the individual’s own classification of being retired. Twenty-eight adults (15 women) aged 55–67 years (mean age 61 years; SD = 2.79) were interviewed. At the time of interview 13 participants were employed but had plans to retire in the next 24 months (mean time until retirement = 11 months, range 1–24 months) and 15 participants had already retired within the last 24 months (mean time since retirement = 10 months, range 1–18 months). The majority of the sample were recruited via community adverts and by word of mouth (n = 19) whilst the rest were recruited via voluntary organisations (n = 9). SES was assigned to participants using the 2010 English Index of Multiple Deprivation (IMD). IMD scores at the Lower Super-Output Area (LSOA) level were obtained from the Office for National Statistics (ONS) website (http://www.ons.gov.uk) for each participant, matched to their home postcode. IMD scores were assigned to deciles numbered from 1–10; decile 1 represents the most socio-economically deprived 10% of LSOAs and decile 10 represents the least deprived 10% of LSOAs in England. Participants’ pre-retirement occupation was categorised according to one of the nine categories within the ONS standard occupational classification [25]. The sample represented a range of SES (IMD range 1–10) and occupational backgrounds (seven of the nine main ONS occupational types including (1) managers, directors & senior officials, (2) professional, (3) associated professional & technical, (4) administrative & secretarial, (5) skilled trade, (6) caring, leisure & other services, and (7) sales & customer services).

### Interview protocol

A Theory Domain Interview schedule [20] was developed based on the 12 domains in the TDF in order to elicit perceptions about anticipated or experienced retirement-related changes in PA behaviour. The domains are, ‘knowledge’, ‘beliefs about consequences’, ‘beliefs about capabilities’, ‘skills’, ‘environmental context & resources’, ‘social influences’, ‘memory, attention & decision processes’, ‘behavioural regulation’, ‘emotion’, ‘social or professional role/identity’, ‘motivation & goals’ and the ‘nature of the behaviour’).PA was explicitly defined at the start of each interview to include sport, structured exercise, PA for recreational, occupational or transport purposes, domestic activities, gardening and ‘Do-It-Yourself’ (DIY) home improvement work. Participants were prompted to report details of their current and typical PA behaviour, as well as anticipated or experienced changes in PA behaviour after retirement, including the type, frequency, intensity and duration (the ‘nature of behaviour’). Participants were then prompted to discuss current PA behaviour and retirement-related changes in PA behaviour within each of the remaining explanatory theoretical domains of the TDF, focusing particularly on whether these domains related to positive or negative influences on PA behaviour. The ‘knowledge’ domain was used in the interview as a prompt about the participant’s knowledge of local PA facilities and knowledge of concessionary prices at PA facilities for older or retired adults. The interview protocol was developed collaboratively within the research team and piloted before the study commenced to optimise comprehension. Please see Additional files 1 and 2 for the interview schedule for working and retired participants, respectively.

### Interview procedure

Participants were interviewed once by a member of the research team at their own homes or on the premises of Newcastle University according to their preference with no other individuals present. All interviews were conducted by a young, female doctoral researcher (SM) with a background in psychology and public health research. Prior to the conduct of interviews the researcher received training in qualitative methodology, interviewing skills and in conducting theory domain interviews. There was no relationship between the researcher and participants prior to study commencement. In order to elicit perceived changes between pre- and post-retirement PA behaviour within individuals, the interview was designed to prompt all participants to reflect upon their pre-retirement PA behaviour and their experienced or anticipated post-retirement PA behaviour within each of the theoretical domains. No participants withdrew from the study. The study was approved by the Newcastle University Faculty of Medical Sciences Research Ethics Committee. All participants provided informed, written consent prior to participation and received a £10 gift voucher after completing the interview.

### Analysis

Interviews were audio-recorded, transcribed verbatim, and anonymised. The TDF provided the structure for the interview to ensure an inclusive coverage of potential theoretically-guided explanations for PA behaviour and behaviour change but was not used as an explicit framework to guide the analysis of the data. Instead, Framework analysis was conducted whereby the analysis was guided by the data and the themes that emerged from it allowing for themes not covered within the TDF to be coded if they emerged. Framework analysis involves coding data according to their importance rather than frequency and provides a systematic method for comparing intra- and inter-individual views [26]. Framework analysis was supported by NVivo 10 software, following a 5-step standard procedure [27]. Step 1: A subset of interview transcripts (n = 6) representing a diverse sample of participants were selected for review. Key ideas and recurrent themes, which were reported as influential for PA behaviour, were noted. Step 2: A thematic framework was developed guided by the research aims and included key themes identified in step 1 and themes identified in previous research regarding PA behaviour in retirement [13]. Step 3: The thematic framework was applied in analysis of the whole dataset and underwent refinements in order to respond to emergent and analytical themes. During this process judgements were made about meaning, relevance and importance of each statement in relation to the interview as a whole and with particular emphasis on common and divergent themes in the data. Step 4: Data within each theme were arranged in thematic charts which allowed comparisons within and between the two main subgroups of interest (i.e. retired and working participants). A standard procedure [28] was used to assess and confirm data saturation for the working and retired subgroups separately during this stage. Comparisons were also made between other relevant subgroups of interest (e.g. IMD score, gender, length of time to/from retirement date). Step 5: The final step involved a systematic process of identifying associations, patterns and emergent themes within the data.

### Data coding reliability

The analysis plan was developed and monitored in regular discussions by the whole research team. An initial dataset was coded independently by one researcher (SM), and a subset (n = 2) was discussed in a data clinic with other members of the research team (FFS, MW) to cross-check interpretations of key issues emerging from the data and jointly agree the final coding frame to be applied in the analysis of the remaining data. A separate subset (n = 2) was discussed in a data clinic with another team member (NH) to assess the reliability of codes applied using the final coding frame. This study adhered to the consolidated criteria for reporting qualitative research (COREQ) [29].

## Results

Interviews lasted between 13 and 53 minutes (mean length 28 minutes). The following section provides a description of sample characteristics including retirement circumstances, the nature of current PA behaviour and anticipated or experienced changes in PA after retirement. In addition, convergent and divergent themes which emerged from the data about factors perceived to influence PA change during the retirement transition are presented.

### Retirement circumstances

Participants reported a range of different retirement circumstances. Some retirement trajectories involved leaving paid work and not working again representing an abrupt end, whereas others involved leaving paid work to do part-time paid or voluntary work representing a gradual reduction in working. Thus, some individual’s retirement reflected an abrupt end whereas others represented a gradual reduction in working hours. Some pre-retirement occupations involved employment by businesses or organisations, whereas other individuals were self-employed. The timing of retirement was determined by individual choice for most participants with a small minority confronted with an involuntary retirement due to redundancy or to help care for a partner. For some individuals retirement occurred before the age of eligibility for state pension.

### Nature of current PA behaviour

All participants reported participating in some form of PA at the time of interview. However, both subsamples reported a wide range of PA types, frequencies, intensities and durations ranging from infrequent slow walking to running and cycling most days of the week. The most commonly reported PA pursuit was walking, which occurred during leisure-time in different formats (e.g. walking with a group, walking alone, walking a dog and walking during golf) as well as for transport and, for some working participants, as part of their occupational role.

### Perceived change in PA levels after retirement

When prompted about changes in PA levels pre- to post-retirement, most participants anticipated or experienced an increase in PA levels after retirement, with a minority reporting a decrease, no change or uncertainty about changes in PA levels after retirement. Some participants discussed the changes in PA levels with reference to the opportunities for PA in their pre-retirement occupational role. For example, some participants with sedentary pre-retirement occupations anticipated or experienced increases in PA levels after retirement. In addition, a small number of working participants who reported having a physically demanding occupation anticipated a reduction in PA levels after retirement unless they actively planned to compensate for the loss. However, there were exceptions to this pattern. For example, one working participant who had a sedentary occupation anticipated a potential net decrease in PA levels after retirement due to the loss of occupational-related transport PA. In addition, some participants who had pre-retirement occupations which may be classified as sedentary reported that their occupation involved a high level of physical activity.

### Factors which influence changes in PA behaviour

Four main themes emerged from the data regarding factors which are perceived to be associated with post-retirement PA behaviour; (1) resources for PA, (2) structure of daily life in retirement, (3) opportunities for PA and (4) transitional PA phases in retirement. Themes are described below and include direct quotes in italics with individual participant characteristics in brackets following each quote. This section presents convergent themes about PA change reported by working and retired participants. Divergent views are emphasised where they occurred. Themes are inclusive of all data and therefore represent the views of all participants within the sample.

### Resources for PA

Participants reported changes in PA after retirement which related to internal and external resources for PA.

#### Time availability

The majority of the participants reported an increase in time availability after retirement which was perceived as a key factor facilitating PA behaviour. Some participants reported that PA was not prioritised during the limited amount of free time after the working day. In fact, participants often reported that they would use available time to achieve other goals which were given higher priority such as domestic chores. Although the increase in time availability after retirement was a commonly perceived positive change facilitating PA behaviour, having more time could also lead to procrastination and taking longer to do PA.

*“Being at work you’ve got to squeeze physical activity in whereas when I will be retired it will be ‘ah I got time to do that’” (Female, 62, IMD 1, working, 6 months until retirement).*

*“You don’t want to spend your time doing [PA] because you’ve got such a small amount of time” (Female, 61, IMD 7, retired, 8 months since retirement).*

*“It’s just work was stopping [PA], because at the weekends I just had my garden to do but when I did the gardening and washing, I lived on my own you see so looked after domestic chores, there wasn’t time left to do much else, life just got in the way really” (Male, 67, IMD 7, retired, 18 months since retirement).*

*“Because you’ve got all the time in the world you think ‘oh well tomorrow will do’, my friend said the same thing but she said tomorrow never comes!” (Female, 62, IMD 3, retired, 6 months since retirement).*

#### Energy levels

Participants perceived tiredness as an additional inhibiting factor for PA behaviour whilst working, even if time was available after work. Retired participants, but not working participants, reported that leaving work was associated with having more energy which facilitated PA behaviour in retirement.

*“When you come in from work after you’ve been working all day you’re too tired to go out” (Female, 60, IMD 5, working, 18 months until retirement).*

*“At the weekends when I had time I was too tired really to do [PA], so since I’ve retired its changed enormously and I’m now doing things that I used to do probably three or four years ago” (Female, 61, IMD 7, retired, 8 months since retirement).*

*“Because it was a very physically and mentally demanding job I didn't really have the time or the energy… I do believe I’m more energised since I finished work because it was very challenging mentally and physically and I don’t have that anymore so I can, you know, I can divert all those energies elsewhere” (Female, 61, IMD 4, retired, 13 months since retirement).*

#### Financial resources

Most participants acknowledged a reduced income after retirement. Some working participants did not perceive financial changes as a major barrier for PA behaviour in retirement because they had a preference for outdoor pursuits such as walking and cycling which have minimal costs. Participants with a wide range of IMD scores reported an increased ‘awareness’ of the cost of PA after retirement but often reported that this was not perceived to inhibit them from doing PA. However, for a few participants the cost of PA did become more important after retirement. One participant believed that concessionary discounts for retired people would compensate for the potential impact of a reduced income. Some working participants considered the idea of being eligible for concessionary prices at PA facilities to be an additional encouragement to engage in PA after retirement. Concessionary prices for public transport were reported as important for enabling retired participants to access attractive environments where they could do PA.

*“I think it will be ok for us, put it this way, I’m not worried about money retiring, I can’t see my standard of living dropping that much” (Male, 64, IMD 2, working, 24 months until retirement).*

*“It might affect how often I get a new pair of trainers or something but nothing major” (Male, 62, IMD 6, working, 8 months until retirement).*

*“For me I don’t think it [reduced income] will [affect PA] but as I said I will be far more conscious of the costs…you perhaps think twice, do I really want to do this, you know, ‘is this a sort of good commitment?’” (Male, 58, IMD 10, working, 3 months until retirement).*

*“You are more acutely aware of managing a limited resource, I think, when you’re not actually earning anything” (Male, 60, IMD 2, retired, 18 months since retirement).*

*“Ok, yes you’re down onto a pension thing but being retired you should be able to get concessions really… should be no more expensive than it is now” (Female, 62, IMD 1, working, 6 months until retirement).*

*“I haven’t really looked into it (concession price at local PA facility) yet but it means that you can go at a cheaper rate… so it will give me a bit more encouragement” (Female, 60, IMD 5, working, 18 months until retirement).*

*“I’ve got my bus pass now and a metro pass that I pay £25 pound a year for instead of £445 pounds a year so that definitely helps [facilitate PA]… I’ll get on the metro free and I’ll get on the bus at Newcastle, go along to Corbridge and have a lovely walk along the river… so that in itself, you know, it opens everything up” (Female, 61, IMD 4, retired, 13 months until retirement).*

### Changes in daily structure

#### Loss of daily structure

One participant attributed her decrease in PA level after retirement mainly to the loss of structure to her day. Anticipated loss of structure tended to be a significant concern for working participants when asked about their PA after retirement. This tended to relate to the likelihood of engaging in sedentary behaviour instead of PA behaviour. The anticipation of losing the structure of the working day raised concerns about motivation for PA and the importance of self-discipline after retirement. However, not all participants perceived major consequences associated with loss of daily structure after leaving work. For example, one self-employed participant did not perceive structure loss to be a negative factor for his PA. Some retired participants attempted to keep the same structure as they had whilst working helped to overcome the potential impact daily structure loss could have on PA. Engaging in structured or organised PA types before retirement was also perceived as helpful for avoiding potential negative consequences of daily structure loss in retirement.

*“I’m losing the structure of my day so I don’t fit as much in unfortunately… retirement is hard to get used to learn how to structure your days properly” (Female, 59, IMD 8, retired, 8 months since retirement).*

*“If I was completely retiring I could see that there would be a danger that I would sit around reading the paper every morning, and then crosswords, and generally not get out and about whereas I think having work you have to get up, you’ve got to be up and about, I can see why you might sort of drift into the more sedentary lifestyle [after retirement]” (Male, 62, IMD 10, working, 2 weeks until retirement).*

*“I think it will have to be self-discipline… it might be a nice evening and I might have sat watching the television, reading, pottering around and, you know, I’ll have to be thinking to myself, ‘right it’s a nice night go for a walk, go for a walk round’ because it’s very easy not to do it, so I think you need more motivation when you retire” (Female, 66, IMD 9, working, 3 months until retirement).*

*“For me the lack of structure is a slight disadvantage, but it’s not a big, big change from when I was working, but I can see that if you’ve been doing a profession or work of some kind for a number of years and that was structured in a particular way, when that goes you really do have to re-assess and re-think how your days are spent” (Male, 60, IMD 2, retired, 18 months since retirement).*

*“I get up the same time as I did when I went to work I do exactly the same thing the only difference is I go out… to the baths [swimming pool] and come back but I keep that momentum” (Male, 55, IMD 2, retired, 8 months since retirement).*

*“I can see that happening [loosing daily structure after retirement] but because there’s a club nature of the things I do, I know the clubs are on a Monday Tuesday and the dancing on a Friday, you know, so it’s like ok, those are fixed points” (Male, 60, IMD 9, working, 12 months until retirement).*

#### Organising PA in retirement

For working participants, the availability of day time to do PA was a favourable change which was perceived to facilitate PA behaviour because they could re-organise the timing of their PA around other interests.

*“I think ‘oh well I’m going to, tomorrow night I’m going to go for a long walk’ and then something will come on the television and I’ll start watching that but I think that’s normal… perhaps when I am retired I will have during the day to do those things” (Female, 66, IMD 9, working, 3 months until retirement).*

### Opportunities for PA

#### Social context

The change in social context after retirement was reported as a key factor influencing PA behaviour for some female participants. For example, one participant reported the positive influence of like-minded friends approaching retirement at the same time. However, other participants reported how social influences could have a negative impact on PA in regards to having to fit in around other people’s PA preferences and routines after retirement.

*“A lot of my friends are coming up to retirement now and, you know, we’ll be able to meet up and go for walks because most of my friends are the kind of outdoor types” (Female, 59, IMD 6, working, 2 months until retirement).*

*“[Husband will say] ‘oh I don’t want to do that’ [swimming],‘oh what do you wanna [want to] do that for?’” (Female, 62, IMD 1, working, 6 months until retirement).*

*“Before I finished work I was swimming on a more regular basis but I haven’t been and I think that’s because with actually finishing work if I were to go off and do one thing [husband] will say oh should we do something else instead, it is easy to be put off” (Female, 59, IMD 8, retired, 8 months since retirement).*

#### Roles and responsibilities

Some retired participants had voluntary jobs and some perceived these to facilitate their PA behaviour whilst others perceived these to limit PA behaviour. Many working participants reported plans to start voluntary work after leaving employment and perceived these roles to facilitate PA behaviour. Participants also anticipated an increase in familial responsibilities, such as caring for grandchildren after retirement which had potential to increase PA behaviour. Some working participants believed that retirement was an optimal opportunity for them to do other things they had always wanted to do which do not relate to PA.

*“We take people out in wheelchairs and that’s physical…they might want to go down to the coast and go all the way round the coast in their wheelchair and you know you’re humping [pushing] them round and up steps and all sorts” (Male, 55, IMD 2, retired, 8 months since retirement).*

*“With voluntary it involves driving and taking cancer patients to hospital and accompany[ing] them…sitting in the car driving for 2 3 hours or more, it makes me less active” (Male, 61, IMD 6, retired, 2 months since retirement).*

*“Well I would certainly expect that I will take on other roles after retirement, and it’s entirely possible that I will do various forms of part time paid work or part time voluntary work, I wouldn’t anticipate that that would inhibit physical activity it might go along with it quite well because the work itself might be quite physical” (Male, 58, IMD 9, working, 15 months until retirement).*

*“Because the grandchildren don’t live close I would have to get a bus and you know walk to the school and walk them home” (Female, 60, IMD 5, working, 18 months until retirement).*

*“I can’t think of anything at the moment, not that relates to physical activity I mean there are other things I might do like learn a language” (Male, 58, IMD 9, working, 15 months until retirement).*

### Transitional PA phases after retirement

Participants discussed further transitional phases occurring after the initial retirement transition which involved distinct changes in PA behaviour. Some retired participants were uncertain about how retirement had impacted on PA behaviour because they had not settled into their retirement patterns. Some participants who had been retired for a considerable amount of time also identified being in an early ‘phase’ of their retirement. Some participants who had been retired for some time reflected upon different temporary ‘phases’ which occurred after their retirement. These different phases in retirement were also anticipated by some working participants. PA behaviour was reported to change during these different phases in retirement. For example, one working participant reported a temporary phase after retirement during which he would be waiting for other friends to retire before he could enjoy certain PA types.

*“We’ve been so busy, we’ve been away on holidays, we’ve been up and down and we haven’t really settled into our retirement pattern as it were” (Female, 60, IMD 9, retired, 2 months since retirement).*

*“It’s still relatively early…my lifestyle hasn’t changed because we’ve been very busy” (Male, 60, IMD 9, retired, 6 months since retirement).*

*“As I say it’s really early days for us yet… just finding my feet at the moment” (Male, 55, IMD 2, retired, 8 months since retirement).*

*“The first few months I didn’t do an awful lot because I was busy doing other stuff and socialising…it’s quite nice not to do anything, not to be anywhere so I did luxuriate in that initially” (Female, 61, IMD 4, retired, 13 months since retirement).*

*“Once [friends] are retired as well and have more time then I’ll be, the motivation to go out and play golf will be greater” (Male, 60, IMD 7, working, 24 months until retirement).*

*“I haven’t decided that gardening and walking are my things forever it’s just that they’re the things that I’m doing at the moment so I think I’m very open to having, you know, doing something else” (Female, 61, IMD 7, retired, 8 months since retirement).*

*“I think early retirement, it’s going to be a lot of decorating and you know tidying drawers, throwing out of things, things like that” (Female, 62, IMD 1, working, 6 months until retirement).*

#### Pre-retirement phases

There was uncertainty about how retirement may impact on physical activity reported by some working participants who were not retiring for some time.

*“You’ve just given me thought there to think about actually, I haven’t really given that a thought to be honest… it’s rather big question that to be quite honest, stirred my imagination a bit” (Male, 64, IMD 2, working, 24 months until retirement).*

*“I’ve never sorta [sort of] given it conscious thought what the hell I’m going to do when I retire but now that you’ve put it in my mind I think it is something that I’m going to have to do, because if I’m sitting watching the tele [television] all day and I have been relatively active [during pre-retirement employment], as I say on my feet all day going about the shop floor is physically tiring to be honest, it’s demanding, so I need something to fill the void, but I’ve never really put much thought as to what that will be” (Male, 62, IMD 6, working, 24 months until retirement).*

## Discussion

### Main findings

The findings of this theory-based interview study provide important insights into the perceptions and experiences of PA change among individuals within the transition from employment to retirement. A number of inter-related factors were sensitive to change during the retirement transition. Individuals perceived a reduction in common barriers to PA such as lack of time, energy and goal conflict after retirement. The cost of PA after retirement was perceived as important for some individuals. However, having time to research the most affordable PA classes and taking advantage of concessions may help to mitigate cost concerns. Although individuals generally perceived an increase in the availability of resources which facilitated PA in retirement, the loss of daily structure after leaving work had negative consequences for PA behaviour among some individuals. More specifically, lack of structure led to procrastination and some individuals emphasised the need for a higher level of motivation and self-discipline to ensure that preferences for other, more sedentary pursuits did not distract them from PA. Those who engaged in structured PA sessions before retirement were less likely to experience these negative consequences after retirement. Conscious efforts to create a similar daily structure in retirement to that whilst working may help to maintain PA levels. Retirement appears to be associated with a number of new opportunities, some of which indirectly facilitate or conflict with PA behaviour. For example, the exposure to a different social context and the adoption of roles and responsibilities may help or hinder PA behaviour in retirement for some individuals. Personal aspirations, purpose-seeking and priorities in retirement may determine whether or not new opportunities influence PA behaviour. Finally, some individuals experienced a number of distinct PA phases in retirement which have differential effects on PA.

### Strengths and limitations

The study examined the subjective experience of working and retired participants to gain a deeper understanding of how PA is perceived to change during the retirement transition. The study utilised a theoretical framework covering all current hypotheses for behaviour and behaviour change. Therefore, the findings can be interpreted in line with theory. Using theory to inform the development of behaviour change interventions is widely recommended [27]. In contrast to previous studies investigating PA in retirement, this study recruited and explored the views and experiences of individuals who were close to retirement or recently retired. The interview schedule also included open-ended questions which elicited rich, descriptive data. The retirement circumstances reported by participants were heterogeneous and reflected a contemporary picture of retirement [30]. Framework analysis enabled the comparison of sub-groups to determine whether perceived PA changes differed as a function of anticipation or experience of retirement.

However, some limitations of the research are acknowledged. Comparative views between working and retired participants are cross-sectional. In addition, the personal and professional characteristics of the interviewer can influence the collection and interpretation of qualitative data [31]. Thus, research findings have been described as a joint product of the participant, the researcher and the relationship between the two parties [32]. To reduce the potential for researcher characteristics to bias data collection, a reflexive interviewing style was adopted. This involved rephrasing questions when there were issues with comprehension, ensuring participants were given sufficient time to think about their responses, and assuring participants there were no right or wrong responses. These strategies can also reduce the likelihood of participants giving stereotypical or socially desirable answers when they do not have any pre-formulated views on the interview topics. The interviewer informed participants that she was interested in views and experiences of PA during the retirement transition and participants seemed at ease providing the researcher their views on this topic. To address the potential for the researcher’s experience to bias the selection and interpretation of data and to ensure that multiple and alternative interpretations were considered, two data analysis clinics were held where the selection and interpretation of the data was scrutinised by members of the research team. Returning the transcripts to participants for checking and eliciting feedback from participants on the findings may have further reduced potential for bias in the selection and interpretation of data. The value of the factors presented as predictors of PA change may be limited because subjective perceptions of how PA and PA determinants change after retirement may not correspond with actual changes. However, eliciting subjective perceptions can illustrate areas where opportunities exist to encourage individuals to increase or maintain PA behaviour during the retirement transition. The current evidence base for the predictive and potentially causal determinants of PA supports the assumption that interventions effectively targeting predictive cognitions can produce PA changes. The sample was relatively small which may limit the generalisability and transferability of the findings to other individuals or populations. However, the varied sample, including pre-retirement and post-retirement individuals, revealed novel insights into how PA is perceived to change after retirement.

### Relationship to previous research

This is the first study to adopt an inclusive theoretical approach to understanding PA change during the retirement transition. Only a limited number of theory-based determinants have been examined as potential candidates explaining PA behaviour in previous studies [15,16]. This is problematic because theories represent the proposed causal determinants of behaviour and therefore provide mechanisms responsible for behaviour that can be targeted during intervention. Furthermore, previous studies have focused on exploring the motivators and barriers to PA in individuals who have retired [15,16] and less on PA determinants which may be sensitive to change during the transition from employment to retirement. As a result, previous studies reveal little about the specific opportunities to promote PA during the retirement transition.

To date, conclusions about PA behaviour in retirement have been based on retrospective reporting in samples of participants who have been retired for some time [13,15,16,33,34]. Retrospective reports may be prone to recall bias and may not accurately capture all important influences of behaviour at the time of the transition. These studies also assume static PA determinants in retirement and fail to capture the dynamic nature of PA during the course of retirement. The findings from the study suggest PA behaviour can change during and up to two years after the initial retirement event. It is important to explore the views of individuals who are approaching retirement and have recently retired. Individuals at later stages of retirement, as those included in previous studies, may have already settled into a more habitual pattern of behaviour and may not be as receptive to programmes aiming to change their PA behaviour. Furthermore, participant samples in previous studies have been biased towards the inclusion of healthy retired individuals who have a high SES, engage frequently in PA behaviour and have exceptionally high levels of motivation for PA behaviour [15,16,33,34]. This study explored a range of views by including a diverse sample of participants at different stages during the retirement transition, with a range of SES and occupational backgrounds and different levels of PA behaviour and motivation for PA behaviour at the time of the interview.

The findings from this study suggest that, for the majority of individuals, retirement may be associated with favourable circumstances which promote the adoption or increased engagement of PA. Based on these findings, PA levels may be predicted to increase around the retirement period for many people; however, studies assessing objectively measured PA in discrete age groups do not appear to provide evidence to confirm these findings but rather a gradual decline in PA is identified with retirement [5]. Previous research has shown that levels of regular, sustained PA decreases during late adulthood [35]. It is possible that the perception of favourable changes within the transition from employment to retirement, are temporal and short-lived. For example, the perception of time availability, increased energy levels, decreased goal conflict and new opportunities for PA may diminish rapidly as soon as an individual has filled up their post-retirement schedule.

It appears that the inconsistent findings regarding the direction of PA change in previous studies may be explained by the considerable intra-individual variability in retirement-related lifestyle changes. Previous research has identified that SES indicators, such as the distinction between manual or non-manual occupations, may be key moderators of the relationship between retirement and PA levels [9,12,36]. In this study, some individuals with sedentary occupations anticipated a decrease in PA levels, which is not in line with previous predictions [12]. The findings from this study suggest that several positive and negative inter-related factors may influence PA levels after retirement, which are not explained by previous occupation alone. For example, being exposed to a different social context after retirement can have an impact on PA behaviour, particularly in respect to the attitudes and preferences of family members. Similar findings have been reported in a recent study exploring how couples influence each other’s physical activity behaviour in retirement [37].In addition, the structure of pre-retirement PA may be a key moderator of the relationship between retirement and PA.

Interviewing participants who were at different stages of the retirement transition revealed that retirement may involve a series of different phases which have differential effects on PA behaviour. This finding complements Atchley’s conceptual model describing the adjustment to retirement [38], where retirement is conceptualised as a transitional *process* which involves different distinct phases. More specifically, the model suggests that some individuals are quick to establish a retirement routine after leaving work, whereas others enter either a ‘honeymoon’ phase where they initiate new activities and opportunities or a ‘rest and relaxation’ phase, where they enjoy a temporary period of calmness after leaving work. It appears that these distinct phases may also shape PA patterns during the retirement transition and individuals may find that they take several months to settle into their post-retirement PA patterns.

The findings from this study support the idea that contemporary routes to retirement are highly individualistic [39]. As a result, PA changes are likely to vary considerably from one individual to another. The study revealed that perceptions of PA change during retirement can be extremely diverse and appear to be determined by individual circumstances including the timing of retirement, the reason for retirement and the voluntary or involuntary nature of retirement. The impact of retirement on PA also appears to depend on exclusive preferences and responsibilities after retirement and the way in which individuals prioritise PA against other aspirations for their retirement.

### Implications

The findings from this study offer understanding of how interventions delivered during the retirement transition can be optimised. Retirement may represent an ideal opportunity to promote PA behaviour in older adults because individuals may be particularly motivated and receptive to attempts to change behaviour due to a perceived reduction in common barriers to PA. Attempts to emphasise changes in time availability, energy, increased opportunities through physically active social or voluntary roles and the availability of free or reduced PA types may be most effective to ensure individuals engage in sufficient PA behaviour after retirement. The potential short-term window of opportunity to promote the positive changes facilitating PA behaviour after retirement has important implications for the timing of future PA interventions. PA interventions may need to commence when individuals are approaching retirement and are in the process of planning their post-retirement schedule. At this stage it may be beneficial to emphasise to individuals that they may experience an increase in energy levels to engage in PA after retirement and to encourage individuals to consider how PA fits in with their retirement roles and goals. Previous research has shown that interventions targeting PA behaviour in adults of retirement age are viable and may be more effective if they include components that are personalised to the individual and their environments [40,41]. Designing interventions which are tailored to the specific needs of the individual may be particularly important when targeting behaviour change in retirement due to the heterogeneity of routes to retirement. One-to-one, personalised intervention approaches are likely to be costly if delivered face-to-face. PA interventions which adopt digital platforms may be more cost-effective and have the additional advantage of reaching a greater number of individuals [42,43]. The content and interface of web-based interventions can be adapted and personalised to the characteristics and preferences of the individual. Encouraging findings are emerging about the acceptability and effectiveness of web-based interventions in older adults [44-46].

### Future research

Future research should explore the extent to which perceived changes in PA during the retirement transition reflect objective changes in PA during this period using methodology which focuses on PA change within individuals. Future research should also explore how individuals perceive sedentary behaviour to change during the retirement transition and how sedentary interests fit into their retirement routines and interpret this in line with the findings from the small number of studies which have examined changes in sedentary behaviour during the retirement transition [6,10,11]. It remains unclear how post-retirement PA is maintained into older adulthood. Future research should investigate the maintenance of PA habits developed during the first two years of retirement into older adulthood. Future research should concentrate on developing and evaluating a range of theory- and evidence-based interventions to promote and maintain PA in and beyond the retirement transition. Intervention research should focus on cost-effective personalised delivery modes (e.g. using digital media).

## Conclusions

PA can significantly reduce the risk of morbidity and mortality in older age, yet older adults do not engage in sufficient levels of PA. The retirement transition represents a critical opportunity to intervene to change PA behaviour and, in turn, promote healthy ageing. The findings from this study fill a gap in theoretical knowledge about PA change during the retirement transition and reveal several inter-related factors and individual circumstances which may explain PA change. Each PA retirement pattern is highly individualised and this should be taken into account during the development of interventions targeting this key life transition. The findings from this study generate important hypotheses about how PA and PA determinants change during the retirement transition which can be explored and tested in future intervention research.

## Additional files

Additional file 1:
**Interview schedule for working participants.** Interview schedule based on the 12 domains in the Theory Domain Framework used to elicit perceptions about anticipated retirement-related changes in physical activity behaviour in working participants. Additional file 2:
**Interview schedule for retired participants.** Interview schedule based on the 12 domains in the Theory Domain Framework used to elicit perceptions about experienced retirement-related changes in physical activity behaviour in retired participants.
