# Supplementary material for: Changes in physical activity during the retirement transition: a theory-based, qualitative interview study
Source: Int J Behav Nutr Phys Act. 2015 Feb 21;12:25. doi: 10.1186/s12966-015-0186-4 (PMC4343052; doi:10.1186/s12966-015-0186-4)
Supplement: Additional file 1: — Interview schedule for working participants. Interview schedule based on the 12 domains in the Theory Domain Framework used to elicit perceptions about anticipated retirement-related changes in physical activity behaviour in working participants. [file 12966_2015_186_MOESM1_ESM.docx]

**Additional file 1. Interview schedule for working participants**

Thank you for agreeing to be interviewed. We are interested in how people view physical activity when they are approaching retirement or when they have recently retired and I would like to ask you some questions about this.

Physical activity refers to all kinds of activities like sports, structured exercise classes or even physical activity for transport such as walking or cycling, as well as DIY and gardening.

There are no right or wrong answers; we are interested in all types of views about physical activity and retirement. If I ask you a question you haven’t thought about feel free to take a few minutes to think about it. If you would rather not answer the question that is no problem, we will move on.

Firstly I’m going to ask you a few questions about you and your circumstances before I go on to ask more detailed questions.

1. Are you retired at the moment?
2. When will you retire?
3. What is your occupation? Is this full time or part time?
4. What age are you?
5. Can you tell what physical activity you are doing at the moment, if any? How often do you do X?
6. How do you feel about the amount of activity you are doing, are happy with it or do you feel you should be doing a bit more?

If participant would like to increase: What would your ideal level of physical activity would be? What types of physical activity would you do if you were to increase your physical activity level?

1. How do you think your physical activity will change after the first year of retirement, if you think it will change at all?

Thinking about your current level of physical activity/ your ideal level of physical activity, I am now going to ask you some specific questions about your physical activity and I am really interested in how you think retirement plays a role...

**Beliefs about capabilities**

1. Can you tell me how easy it is for you to be physically active at the moment?

2. What have you encountered that makes it difficult, if anything?

3. Are there any health issues that have got in the way of your physical activity?

4. What would or has helped you overcome these difficulties?

5. On the other hand what have you encountered that makes it easy, if anything?

6. In what way has X helped?

*Prompts about retirement*

7. How easy or difficult do you think it will be to be physically active after you retire?

8. Do you expect any of the issues we have just discussed to change after retirement?

**Beliefs about consequences**

1. What would you say are the advantages of being physically active?

2. What would you say are the disadvantages of being physically active?

3. Would you say the advantages outweigh the disadvantages?

*Prompt about retirement:*

4. Do you see these advantages or disadvantages of being physically active changing after you retire?

**Social influences**

1. What do you think your family and friends would think of you being physically active?

2. How much physical activity do you think other men/women the same age as you are doing?

3. Would you say this influences what you do?

4. Do you have someone who encourages or supports you to be physically active? How do they encourage/support you?

*Prompts about retirement:*

5. We spoke about how much other people’s thoughts or behaviours may or may not influence what you do. How do you think this will change after retirement? For example, do you think you will be more or less likely to be influenced by others after retirement or will it stay the same? If so, in what way will it change?

**Knowledge**

1. What do you know about the physical activity facilities that are available to you in your area?

2. Are there any reduced prices for access to these facilities that you know about for someone who is older or retired?

*Prompt about retirement*

3. Do you think the availability of concessions will influence your physical activity after you retire?

**Environmental context & resources**

1. Is there anything in your surroundings that you think helps you to be physically active? For example, would you say the area you live in provides opportunities to be active? If so, in what way does it provide opportunities? (Prompt about a range of ‘facilities’ including gyms, swimming pools and outdoor green space)

2. On the other hand, is there anything about your surroundings that you think stops you from being physically active?

3. What about resources like money or access to facilities or equipment for physical activity, can you tell me if and how this influences your physical activity? (Prompt about money, access to facilities, equipment)

*Prompt about retirement*

4. Do you think retirement will have any impact on the resources we have talked about? (Prompt about money, access to facilities, equipment). If so, how do you think changes in X will affect your physical activity, if at all?

**Motivation & goals**

1. Can you tell me a bit about whether you feel motivated to be physically active?

2. What would you say is your main motivation to be physically active?

3. Are there any other things that you like to do that get in the way of being physically active?

4. Are there any other things that you need to do that get in the way of being physically active?

*Prompt about retirement*

5. Can you see your motivation levels changing after retirement? If so, in what way will it change?

6. Do you think there will be other things you want to do or need to do that will interfere with physical activity after retirement?

**Behavioural regulation**

1. Can you tell me about how you organise your physical activity? For example, do you plan ahead or have a set routine?

*Prompt about retirement*

2. Do you think the way you organise your physical activity will change after retiring? For example, do you think you will plan your physical activity more or less?

**Emotions**

1. Can you tell me what your mood is like after being physically active? For example, are you likely to feel happy? Energetic? Exhausted?

2. Would you say you are more or less physically active depending on the mood you are in at the time?

*Prompt about retirement*

3. Do you think any of this will change after you retire? For example, will mood play a role to the same extent after retirement?

**Memory, attention and decision processes**

1. When people retire they often say that they lose the structure in their day, do you think that means it can be easy to forget to be physically active?

2. Is there anything that distracts you and then makes you forget to be physically active?

*Prompt about retirement:*

3. Compared to now, how likely is it that you will forget to be physically active or be distracted by other things after you retire?

**Social or professional role/identity**

1. Would you say that being physically active is part of your personality or who you are?

*Prompts about retirement:*

2. After leaving work some people take on other ‘roles’ and these roles may be associated with changes in physical activity. For example, some people look after their grandchildren more, whereas others become formal or informal carers or take on different types of voluntary work. Are there any roles that you will have after retirement?

3. How do you think this role will influence your physical activity? Do you think it will help you or stop you from being physically active? In what way?

**Skills**

1. At the moment, would you say there are any skills you would like to learn that would help you to be physically active? For example, some people have mentioned that they would like to learn to swim or ride a bike?

*Prompt about retirement:*

2. Is this something you are planning to do in retirement?

Thank you very much, that is all my questions. But is there anything else you would like to add that we maybe haven’t covered?

Thanks again for your time.
