# Supplementary material for: Changes in physical activity during the retirement transition: a theory-based, qualitative interview study
Source: Int J Behav Nutr Phys Act. 2015 Feb 21;12:25. doi: 10.1186/s12966-015-0186-4 (PMC4343052; doi:10.1186/s12966-015-0186-4)
Supplement: Additional file 2: — Interview schedule for retired participants. Interview schedule based on the 12 domains in the Theory Domain Framework used to elicit perceptions about experienced retirement-related changes in physical activity behaviour in retired participants. [file 12966_2015_186_MOESM2_ESM.docx]

**Additional file 2. Interview schedule for retired participants**

Thank you for agreeing to be interviewed. We are interested in how people view physical activity when they are approaching retirement or when they have recently retired and I would like to ask you some questions about this.

Physical activity refers to all kinds of activities like sports, structured exercise classes or even physical activity for transport such as walking or cycling, as well as DIY and gardening.

There are no right or wrong answers; we are interested in all types of views about physical activity and retirement. If I ask you a question you haven’t thought about feel free to take a few minutes to think about it. If you would rather not answer the question that is no problem, we will move on.

Firstly I’m going to ask you a few questions about you and your circumstances before I go on to ask more detailed questions.

1. Are you retired at the moment?
2. When did you retire?
3. What was your occupation? Was this full time or part time?
4. What age are you?
5. Can you tell what physical activity you are doing at the moment, if any? How often do you do X?
6. How do you feel about the amount of activity you are doing, are happy with it or do you feel you should be doing a bit more?

If participant would like to increase: What would your ideal level of physical activity would be? What types of physical activity would you do if you were to increase your physical activity level?

1. How do you think your physical activity changed after the first year of retirement, if you think it did change?

Thinking about your current level of physical activity/ your ideal level of physical activity, I am now going to ask you some specific questions about your physical activity and I am really interested in how you think retirement plays a role...

**Beliefs about** **capabilities**

1. Can you tell me how easy it is for you to be physically active at the moment?

2. What have you encountered that makes it difficult, if anything?

3. Are there any health issues that have got in the way of your physical activity?

4. What would or has helped you overcome these difficulties?

5. On the other hand what have you encountered that makes it easy, if anything?

6. How would you say X has helped?

*Prompt about retirement*

7. How easy or difficult do you think it is to be physically active now that you have retired?

8. Have any of the issues we have just discussed changed after retirement?

**Beliefs about consequences**

1. What would you say are the advantages of being physically active?

2. What would you say are the disadvantages?

3. Would you say the advantages outweigh the disadvantages of being physically active?

*Prompt about retirement*

4. Would you say the advantages or disadvantages of being physically active changed after retirement?

**Social influences**

1. What do you think your family and friends would think of you being physically active?

2. How much physical activity do you think other men/women the same age as you are doing?

3. Would you say this influences what you do?

4. Do you have someone who encourages or supports you to be physically active? How do they do they encourage/support you?

*Prompt about retirement*

6. We spoke about how much other people’s thoughts or behaviour influence what physical activity you do. Do you your thoughts about this has changed after retirement in any way? For example, do you think you are more or less likely to be influenced by others after retirement or has that not changed? If so, in what way has it changed?

**Knowledge**

1. What do you know about the physical activity facilities that are available to you in your area?

2. Are there any reduced prices for access to these facilities that you know about for someone who is older or retired?

*Prompt about retirement*

3. Do you think the availability of concessions has or will influence your physical activity now that you have retired?

**Environmental context & resources**

1. Is there anything in your surroundings that you think helps you to be physically active? For example, would you say the area you live in provides opportunities to be active? If so, in what way does it provide opportunities? (prompt about a range of ‘facilities’ including gyms, swimming pools and outdoor green space)

2. On the other hand, is there anything about your surroundings that you think stops you from being physically active?

3. What about resources like money or access to facilities or equipment for physical activity, can you tell me if and how this influences your physical activity levels? (Prompt about money, access to facilities, equipment)

*Prompt about retirement*

4. Would you say any of the resources we have mentioned have changed since retirement? (Prompt about money, access to facilities, equipment). If so, how did the change in X affect your physical activity if it did?

**Motivation & goals**

1. What about motivation, can you tell me a bit about whether you feel motivated to be physically active?

2. What would you say is your main motivation to be physically active?

3. Are there any other things you like to do that get in the way of being physically active?

4. Are there any things that you need to do that get in the way of being physically active?

*Prompt about retirement*

5. Would you say your motivation levels have changed in any way since retirement? If so, in what way have your motivation levels changed?

6. Were there any other things you wanted to do or needed to do that interfered with your physical activity before you retired?

**Behavioural regulation**

1. Can you tell me about how you organise your physical activity? For example, do you plan ahead or have a set routine?

*Prompt about retirement*

2. Do you think the way you organise your physical activity has changed since you retired? For example, do you plan your physical activity more or less now?

**Emotions**

1. Can you tell me what your mood is like after being physically active? For example, are you likely to feel happy? Energetic? Exhausted?

2. Would you say you are more or less physically active depending on the mood you are in at the time?

*Prompt about retirement*

3. Do you think your mood before or after physical activity is different now that you have retired? For example, does mood play a role to the same extent?

**Memory, attention and decision processes**

1. When people retire they often say that they lose the structure in their day, do you think that means it can be easy for you to be physically active?

2. Is there anything that you find distracts you and then makes you forget to stay active?

*Prompt about retirement:*

3. In comparison to before you retired, how likely is it that you will forget to be physically active or be distracted by other things now that you have retired? Has this changed in any way?

**Social or professional role/identity**

1. Would you say that being physically active is part of your personality or who you are?

*Prompt about retirement:*

2. After leaving work some people take on other ‘roles’ and these roles may be associated with changes in physical activity. For example, some people look after their grandchildren more, whereas others become formal or informal carers or take on different types of voluntary work. Are there any roles that you now have since retiring?

3. How does you think this role influences your physical activity? Do you think it helps you or stops you from being physically active in any way?

**Skills**

1. At the moment, would you say there are any skills you would like to learn that would help you to be physically active? For example, some people have mentioned that they would like to learn to swim or ride a bike?

*Prompt about retirement:*

2. Is this something you are planning to do in retirement?

Thank you very much, that is all my questions. But is there anything else you would like to add that we maybe haven’t covered?

Thanks again for your time.
